# Artificial Intelligence Algorithms for Malware Detection in Android-Operated Mobile Devices

**DOI:** 10.3390/s22062268

**Published:** 2022-03-15

**Authors:** Hasan Alkahtani, Theyazn H. H. Aldhyani

**Affiliations:** 1 College of Computer Science and Information Technology, King Faisal University, P.O. Box 400, Al-Ahsa 31982, Saudi Arabia; hsalkahtani@kfu.edu.sa; 2 Applied College in Abqaiq, King Faisal University, P.O. Box 400, Al-Ahsa 31982, Saudi Arabia

**Keywords:** android applications, malware, machine learning, deep learning, cybersecurity

## Abstract

With the rapid expansion of the use of smartphone devices, malicious attacks against Android mobile devices have increased. The Android system adopted a wide range of sensitive applications such as banking applications; therefore, it is becoming the target of malware that exploits the vulnerabilities of the security system. A few studies proposed models for the detection of mobile malware. Nevertheless, improvements are required to achieve maximum efficiency and performance. Hence, we implemented machine learning and deep learning approaches to detect Android-directed malicious attacks. The support vector machine (SVM), k-nearest neighbors (KNN), linear discriminant analysis (LDA), long short-term memory (LSTM), convolution neural network-long short-term memory (CNN-LSTM), and autoencoder algorithms were applied to identify malware in mobile environments. The cybersecurity system was tested with two Android mobile benchmark datasets. The correlation was calculated to find the high-percentage significant features of these systems in the protection against attacks. The machine learning and deep learning algorithms successfully detected the malware on Android applications. The SVM algorithm achieved the highest accuracy (100%) using the CICAndMal2017 dataset. The LSTM model also achieved a high percentage accuracy (99.40%) using the Drebin dataset. Additionally, by calculating the mean error, mean square error, root mean square error, and Pearson correlation, we found a strong relationship between the predicted values and the target values in the validation phase. The correlation coefficient for the SVM method was R^2^ = 100% using the CICAndMal2017 dataset, and LSTM achieved R^2^ = 97.39% in the Drebin dataset. Our results were compared with existing security systems, showing that the SVM, LSTM, and CNN-LSTM algorithms are of high efficiency in the detection of malware in the Android environment.

## 1. Introduction

In recent years, the popularity of the Android operation system has attracted the attention of malware developers, whose work has grown rapidly [1,2]. Many malware developers focus on hacking mobile devices and changing them into bots. This allows hackers to access the infected device and other connected devices and form botnets. Botnets are used to execute different malicious attacks, such as distributed denial-of-service (DDoS) attacks, sending spam, data theft, etc. The malicious botnet attacks are developed with advanced techniques (e.g., multi-staged payload or self-protection), making it difficult to identify the malware. This, in turn, poses major threats that require the design of effective approaches to detect these attacks [3].

**Figure 1:**
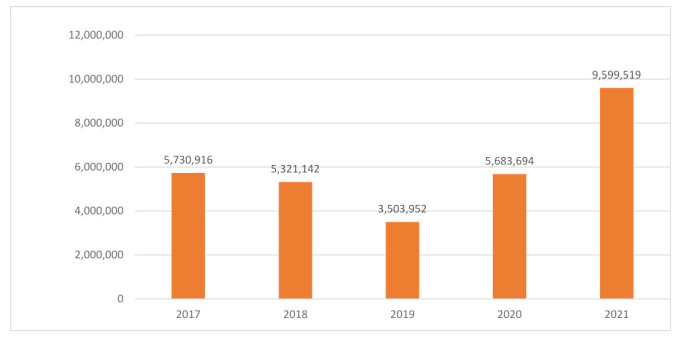
Malware installation packages for smartphone devices.

Android botnets are used to perform attacks on the targeted devices. DDos attacks are achieved by flooding the target machine with superfluous requests and blocking legitimate requests, thus, causing a failure of the targeted system and disruption of the services [4]. Consequently, to protect against such attacks, machine learning methods are proven to be effective in detecting and tracking these threats in the internet of things [5,6]. Haystack [7] reported that a third-part of software-development companies manage 70% of the mobile application and control the personal data of users. According to the AV-TEST Security Institute [8], malicious programming increased, with 5.7 million malware Android packages detected by Kaspersky in 2020, three times more than in 2019 (2.1 million). Figure 1 summarize the increase of malware installation packages for smartphone devices in the last five years. Therefore, signature-based malicious installation packages for the extraction of malware patterns relying on their characteristics can be an effective strategy to secure mobile applications.

Malicious attacks occur in different enrolments with a variety of methods such as fuzzing, denial of service, DDoS, port scanning, and probing [9]. These attacks can be threatening to transport, application layers, or different protocols such as internet control message protocol, file transfer protocol, user datagram protocol, simple mail transfer protocol, transmission control protocol, hypertext transfer protocol, etc. Network-based intrusion detection systems can be used to deal with such attacks by scanning the network and detecting them [10]. 

**Figure 2:**
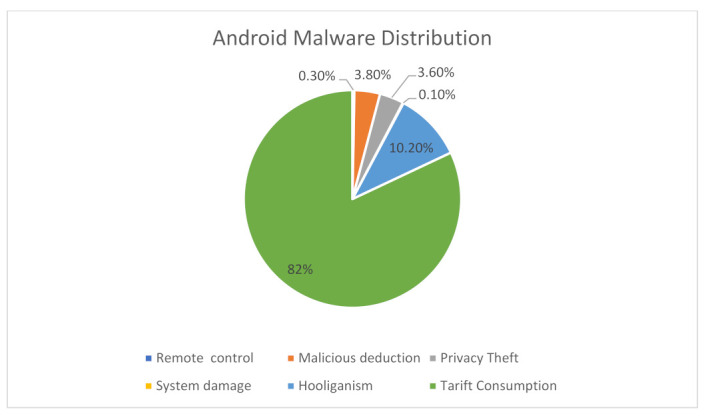
Percentage of Android malware [14].

Usually, in the Android system, security is in-built, where the sandboxing method and permission system are designed to reduce the risk of Android applications [11]. The former was developed using the Linux environment for running Android applications, which allows users to enable permission for the installation of any Android application [12]. However, when updating or upgrading mobile applications, security and privacy features such as time permission, background location, storage, etc., are changed, giving a timeframe for malware attacks. It is possible to exploit Android vulnerabilities during the application developed by users since the Google Play Store cannot detect malicious attacks after the publication of the applications [13]. The percentage of Android malware is presented in Figure 2.

Intrusion detection systems are developed using machine learning and deep learning methods. However, the machine learning technique cannot cope with the huge traffic of data flooding the system. Similarly, deep learning methods fail to provide low generalization errors due to the absence of optimization. Fixed Android botnet datasets make it feasible to design detectors with high detection rates [15], but having complex traffic data hinders the obtention of an accurate prediction rate. This has motivated the development of techniques that are based on Android-malware neuro-evolution classification, thus, providing the number of layers and neurons along with the detection process [16]. 

The present study aimed to extract static and dynamic features from unknown applications; these features show if a particular application is “normal” or “attack”. These features are used to examine the performance of several machine learning and deep learning models, including the k-nearest neighbors (KNN) [17], support vector machine (SVM) [18], convolutional neural networks (CNN) [19], dense neural networks [20], gated recurrent units (GRU), long short-term memory (LSTM) [21], and the hybrid deep learning convolutional neural networks long/short-term memory (CNN-LSTM) and convolutional neural networks/gated recurrent units CNN-GRU [22] methods.

In this study, we investigated and estimated the performance of various machine learning and deep learning algorithms in the detection of mobile malware attacks. This study offers the optimal algorithms for the monitoring of Android applications against malicious attacks. Thus, our research aims to contribute to this field with the following:

1. The development of intrusion detection in the Android system using various machine learning and deep learning algorithms.
2. The proposed system was tested and evaluated using two standard Android datasets.
3. A comparison between the tested algorithms and different state-of-the-arts models is presented.
4. The sensitivity analysis was used to find significant relationships between dataset features and the proposed classes of the datasets.

## 2. Background of Study

This section offers an overview of previous research related to intrusion detection systems, Android malware detection, and standard datasets of Android malicious attacks. Furthermore, it provides an overview of the machine learning and deep learning approaches applied to the design of cybersecurity systems.

The regular improvement of sophisticated Android malware families, e.g., Chamois malware, has made the task of detecting malicious attacks daunting. To tackle this, researchers developed machine learning techniques that improved the available systems. Recently, many studies have applied machine learning models for Android botnet detection, such as linear regression, KNN [23], SVM, and decision trees (DT) algorithms [24]. Some of these recent studies [25,26] used deep learning algorithms, although they do not provide a thorough understanding of their effectiveness. Therefore, the current study compares with deep learning models to examine their effectiveness in Android botnet detection with the use of the available installation support center of expertise (ISCX) botnet dataset [27,28,29].

Kadir et al. [30] used deep learning models to analyze Android botnet attacks in an attempt to understand the latter’s hidden features. The system was evaluated using the ISCX Android botnet dataset, which contained 1929 samples. Anwar et al. [31] proposed an Android botnet detection approach based on static functions. The features of permissions, MD5 signatures, and broadcast receivers were combined and processed with machine learning algorithms. The input data collected from the ISCX dataset were 1400 from different botnet applications, with the system achieving an accuracy of 95.1% in distinguishing Android botnet attacks [32]. 

Several machine learning algorithms were proposed to classify normal and abnormal botnet attacks. In one study, the results indicated that the random forest approach had 0.972% precision and 0.96% recall. In [33], machine learning approaches were proposed for detecting Android botnets. The ISCX dataset consisted of 1635 benign and 1635 attacks. The random forest tree model achieved 97%. In another study [34], the DT, Naive Bayes, and random forest machine learning algorithms were used to detect Android attacks. The information gain method was used to select the significant features. The random forest algorithm achieved a 94.6% accuracy. Karim et al. [35] proposed the static analysis approach to explore the pattern of the features of Android botnet attacks. The features were compared with the intrusion application using the Drebin dataset [36]. Artificial intelligence (AI) approaches using a knowledge-based system were used to secure Android mobiles against malicious attacks [37,38]. Inspired by a meta-heuristic rule and based on fuzzy logic, intrusion detection and data mining systems were developed [39], while machine learning approaches were applied in the development of IDS applications [40,41,42]. The design of IDS systems employed the artificial bee colony [43], particle swarm optimization [44], grey wolf optimization [45], and artificial fish swarm [46] algorithms.

Many systems were developed based on signature-based Android malware detection approaches and behavior-based Android malware intrusion detection approaches [47]. The former is a simple detection method that manages intrusions’ low degree of false positives. The latter is based on anomaly detection and is a very common method using AI algorithms to detect malicious attacks. Numerous research articles aimed to detect and classify Android malware and attacks using machine learning and deep learning approaches, such as the DT and deep learning approaches [48]. By using the generative adversarial networks algorithm [49], it was shown that traditional machine learning was successful in detecting malware in an Android environment [50]. 

Most of the published studies used datasets from Google Play [51], AndroZoo, Android Permission [52], Andrototal [53], Wandoujia [54], Kaggle [55], and CICMaldroid [56]. The present study aimed at developing a system to detect malware attacks in Android environments that have an in-built security system. However, there are still many Android applications with design weaknesses and security flaws that can be threatening to end-users. Therefore, it is crucial to use machine learning and deep learning algorithms to detect Android malware and vulnerability analysis to prevent the development of malware and attacks by hackers [57,58].

## 3. Materials and Methods

**Figure 3:**
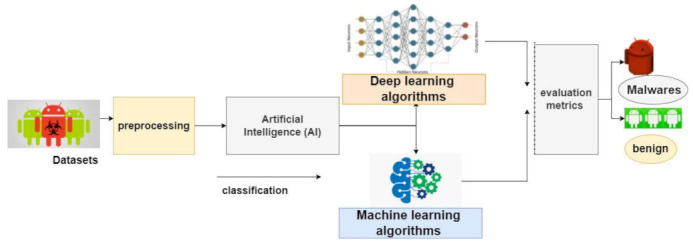
A generic representation of the models applied for the detection of Android malware.

In 2008, Android was developed. With the increasing number of Android applications, companies immediately discussed and built security tools [2]. Nevertheless, the Android system is suffering from security weaknesses. In the last five years, AI approaches focused on protecting the Android system, with many researchers studying the appropriate AI approaches to obtain high accuracy. The framework of the present research is presented in Figure 3. The machine learning algorithms support vector machine (SVM), k-nearest neighbors (KNN), linear discriminant analysis (LDA) and the deep learning algorithms long short-term memory (LSTM), convolution neural network-long short-term memory (CNN-LSTM), and autoencoder algorithms were used to detect malware and attacks against Android applications. These algorithms were tested using two standard datasets. The research questions of this study were:

(1) What are the appropriate machine learning and deep learning algorithms to detect malware in Android?
(2) What are the validation accuracy, robustness, and efficiency of the proposed machine learning and deep learning models related to the detection of Android malware?

### 3.1. Android Dataset

**Figure 4:**
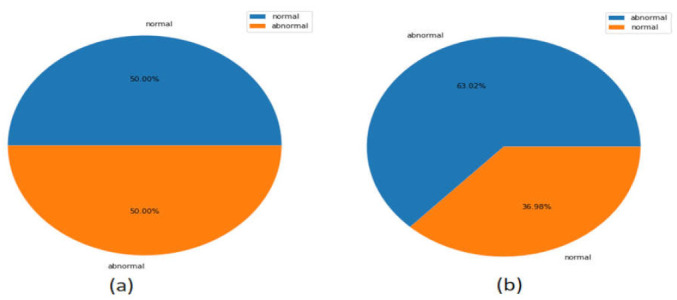
Percentage of classes of the datasets (**a**) CICAndMal2017 and (**b**) Drebin.

The experiments were conducted with two standard datasets: the Canadian Institute for Cybersecurity (CICAndMal2017) and Drebin datasets. The percentage of the classes for the entire CICAndMal201 and Drebin datasets is presented in Figure 4.

#### 3.1.1. CICAndMal2017

The CICAndMal2017 was developed by Canadian Institute; the Cybersecurity dataset is a standard mobile malware dataset containing static and dynamic features of log files. The dataset was generated from 80 network flows using CICFlowMeter-V1 and CICFlowMeter-V3. To examine the proposed system, 667 Android malware packets consisting of 413 features were considered for the injection of malicious and normal packets. The dataset is available from this link: https://www.kaggle.com/saurabhshahane/android-permission-dataset, (accessed on 25 November 2021).

#### 3.1.2. The Drebin Dataset

The Drebin dataset was extracted from 15,037 applications of the Drebin project, which contains 215 features and the injection of 5560 malware and 9476 normal applications. The dataset was developed by the Drebin project and published as the DroidFusion paper in the *IEEE Transactions on Cybernetics* journal [59]. The dataset was generated with different Android applications and is available through the following link: https://www.kaggle.com/shashwatwork/android-malware-dataset-for-machine-learning (accessed on 25 Novmber 2021).

### 3.2. Preprocessing

The Android datasets have different formats and characteristics; therefore, preprocessing is very important for managing the dataset.

#### Min–Max Normalization Method

Normalization is a scaling approach to shift and rescale the values of datasets. The min–max normalization method was applied to scale the data in the range between 0 and 1. The normalization method was applied for the overlap of the entire dataset using the following equation:

(1) $$\overset{´}{V}=\frac{V-x_{min}}{\max\left(A\right)-\min\left(A\right)}\left(\mathrm{new}\_\max\left(A\right)-\mathrm{new}\_\min\left(A\right)\right)+\mathrm{new}\_\min\left(A\right)$$

where, min(A) and max(A) are the minimum and maximum data, respectively, new_min(A) and new_max(A) are the new values of the minimum and maximum used for the scaling of the data, and $\overset{´}{V}$ is the normalized data.

### 3.3. Classification Algorithms

In this section, the theoretical description of the machine learning and deep learning algorithms used in this research is presented.

#### 3.3.1. K-Nearest Neighbors (KNN)

The KNN algorithm is a simple and common machine learning algorithm used to classify numbers of real-life applications by discovering neighbors. The mechanism of the KNN algorithm is finding the distance between the classes of normal values and attacks by selecting object values close to the class k-values. The algorithm starts by loading network data with the length of input data [60]. KNN is utilized to determine the k-values that are near a set of specific values in the training dataset. The majority of these k-values fall into a confirmed class. Furthermore, the input sample is classified. In this research, the Euclidean distance function ($E_{i}$) was used to find the distance between the object values. The expression of the Euclidean distance function is as follows:

(2) $$E_{i}=\sqrt{\left(a_{1}-a_{2}\right)+{\left(b_{1}-b_{2}\right)}^{2}}$$

where a_1_, a_2_, b_1_, and b_2_ are variables of the input data.

#### 3.3.2. Support Vector Machine (SVM)

SVM is a supervised machine learning algorithm developed to solve complex problems in linear and nonlinear applications. It is used to draw the hyperplane between the data points that are near the hyperplane and calculate the effect of the location and orientation of the hyperplane, called the support vector (SV) [61]. The good performance of SV is attained when the distance of the data points is close to the hyperplane. The support vector machine has a number of functions, linear and non-liner; the RBF is appropriate for separable patterns because the network data has a complex format. In this research, a Gaussian radial basis function was proposed to detect Android malware:

(3) $$K\left(y,y^{\prime }\right)=\exp\left(-\frac{{\left|\left|y-y^{\prime }\right|\right|}^{2}}{2\sigma^{2}}\right)$$

where, $y,\;and\;y^{\text{'}}$ are vector features of the training data, $||y-y|{|}^{2}$ is the squared Euclidean distance between the features of the training data, and σ is the parameter.

#### 3.3.3. Linear Discriminant Analysis (LDA)

**Figure 5:**
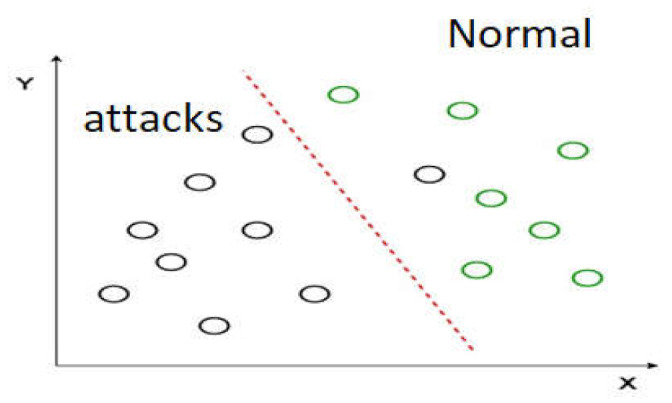
The linear discriminant analysis (LDA) method for analyzing datasets.

LDA is a linear machine learning algorithm used to solve applications with high dimensionality [62]. It is used to model and transform data from a high-space dimension into a low-space dimension by separating the classes of the data into two groups: normal and malicious packets. Figure 5 represent the LDA method for analyzing normal and abnormal packets, where the red line linearly separates the two classes of the data.

#### 3.3.4. Deep Learning Models

**Figure 6:**
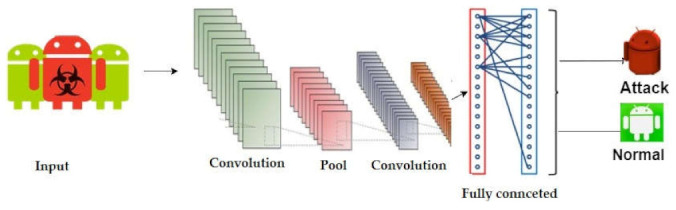
Structure of the CNN model.

CNN-LSTM is a fusion model created with the combination of CNN and LSTM; both are deep learning AI algorithms. In CNN, there are hidden neurons with trainable weights and bias parameters. It is broadly applied to analyze the data in a grid layout, making it different from other structures [63]. It is also called a feed-forward network because the input data stream in one way, from the input to the production layer [64]. Three are the main components in the CNN structure: the convolutional, pooling, and fully connected layers. For feature extraction and the reduction of dimensionality, the convolutional and pooling layers are employed. The fully connected layer is completely folded and attached to the output of the previous layer. The main architecture of the CNN model for detecting Android malware applications is displayed in Figure 6.

**Figure 7:**
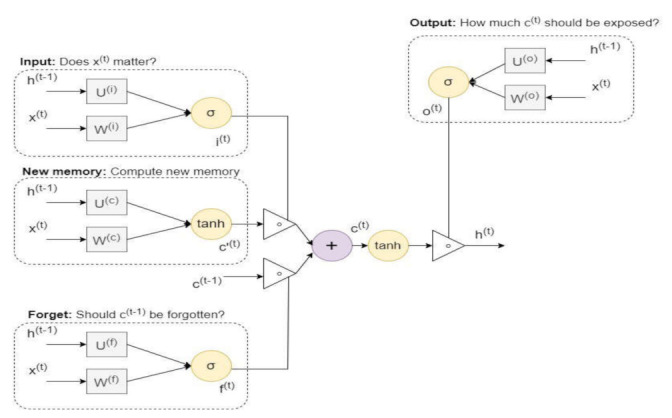
The structure of the LSTM technique.

Hochreiter et al. [65] introduced the LSTM algorithm for learning long-term data dependency. The LSTM is one type of recurrent neural network (RNN). The distinction between the LSTM and RNN techniques is the memory cells present in the LSTM structure. Every memory cell comprises four gates: the input, candidate, forget, and output gates. The forget gate categorizes the input features as to whether they must be discarded or kept. The input gate revives the memory cells in the LSTM structure, and the hidden state is always controlled by the output gate. Furthermore, LSTM uses an embedded memory block and gate mechanism that enables it to address complications related to the disappearing gradient and the explosion gradient present in the RNN learning [66]. The structure of the LSTM model is presented in Figure 7. Table 1 show the parameters of the LSTM model. It is investigated that these parameter values were significant for obtaining high performance to detect the android malware. The kernel size of convolution was 4, the max pool size id 4 for selecting significant features from the filter layer. The drop out value was 0.50 for preventing the model from overfitting; in order to optimize the model, the RSMprop optimizer function is presented. The error gradient is used batch size 150. The equations for the LSTM-related gates are defined as follows:

(4) $$f_{t}=\sigma \left(W_{f\;}.\;X_{t}+W_{f}.\;h_{t-1}+b_{f}\right)$$

(5) $$i_{t}=\sigma \left(W_{i}.\;X_{t}+W_{i\;}.\;h_{t-1}+b_{i}\right)$$

(6) $$S_{t}=\tan h\left(W_{c}.\;X_{t}+W_{c\;}.\;h_{t-1}+b_{c}\right)$$

(7) $$C_{t}=(i_{t}*S_{t}+f_{t}*S_{t-1})$$

(8) $$o_{t}=\sigma \left(W_{o}+X_{t}+W_{o}\;.\;h_{t-1}+\;V_{o}\;.C_{t}+b_{o}\right)$$

(9) $$h_{t}=o_{t}+\tan h\left(C_{t}\right)\;$$

where $X_{t}$ is the vector of the input features sent to the memory cell at a time *t.*$\;W_{i}$, $W_{f}$, $W_{c}$, $W_{o}$, and $V_{O}$ represent the weight matrices, $b_{i}$, $b_{f}$, $b_{c}$, and $b_{o}$ indicate the bias vectors, $h_{t}$ is the point of the stated value of the memory cell at a time *t*, $S_{t}$ and $C_{t}$ are the defined values of the candidate state of the memory cell and the state of the memory cell at time *t*, respectively. *σ* and *tanh* are activation functions, and $i_{t}$, $f_{t},$ and $o_{t}$ are obtained values for the input gate, the forget gate, and the output gate at time *t*, respectively. $h_{t-1}$ represents the short memory vector.

**Figure 8:**
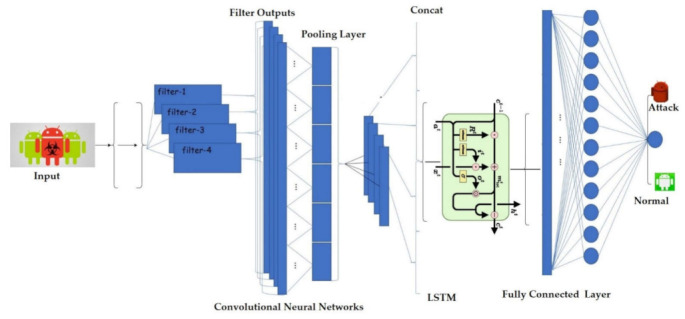
The structure of the CNN-LSTM model.

The CNN-LSTM model was built, as shown in Figure 8. It was trained using the training dataset, and its hyperparameters were adjusted using the Adam optimizer and the validation dataset. The CNN-LSTM model was next implemented on the test dataset, including features of each testing record to its real class: normal or a particular class of attack [67]. The training and optimization processes of the CNN-LSTM model consisted of two one-dimensional convolution layers that cross the input vectors with 32 filters and a kernel size of 4, two fully connected dense layers composed of 256 hidden neurons, and an output layer that applies the nonlinear SoftMax activation function used for multiclass classification tasks. To overcome the model’s overfitting, the global max-pooling and dropout layers were applied. The global max-pooling layer prevents overfitting of the learned features by captivating the maximum value, while the dropout layer is used to deactivate a set of specific neurons in the CNN-LSTM network. The Adam optimizer updates the weights and improves the cross-entropy loss of function. Table 2 show the parameters of the CNN-LSTM model.

#### 3.3.5. Autoencoder (AE)

**Figure 9:**
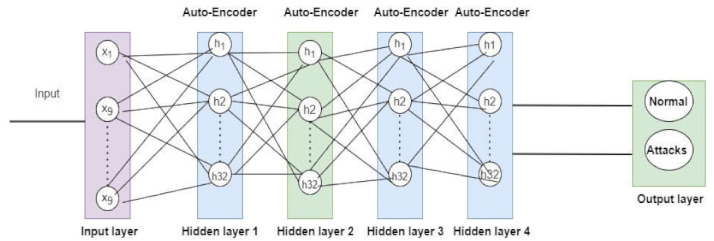
The structure of the auto-encoder (AE) model.

AE is a type of AI algorithm based on deep neural networks that use unsupervised learning for encoding and decoding the input data and are commonly utilized for feature extraction and denoising [68]. Two different processes are performed by AE: encoding and decoding. Hence, its structure is symmetrical. The input data are passed through three different layers: the input, latent, and output layers. These layers make up the AE architecture (Figure 9). The input and output layers have the same size, and the latent layer has a smaller size than the input layer [69]. Encoding and decoding are achieved with the following equations, respectively:

(10) $$e=f_{\theta }\left(x\right)=s\left(Wx+b\right)$$

(11) $$\tilde{x}=g_{\;\theta \;\text{′}}\left(e\right)=s\left(W^{\prime }e+b^{\prime }\right)$$

where *x* is the input vector, *e* ∈ [0, 1] d represents the latent vector, and $\tilde{x}$ ∈ [0, 1] D is the produced vector. From the input layer to the latent layer, the encoding process is repeated. Next, the decoding process is repeated from the latent layer to the output layer. W and $W^{\prime }$ represent the weight from the input to the latent and from the latent to the output layers, respectively. b and $b^{\prime }$ denote the bias vectors of the input layer and the latent layer. The activation functions of the latent layer neurons and the output layer neurons are represented with $f_{\theta }$ and $g_{\;\theta \;\text{′}}$, respectively. The weight and bias parameters are learned in the AE structure after reducing the reconstruction error. Equation (12) is used to measure the error between the reconstructed $\tilde{x}$ and the input data x for individual instances:

(12) $$J\left(W,b^{\prime },\;x,\;\tilde{x}\;\right)=\frac{1}{2}\;\|h_{w,b}\left(x\right)-\tilde{x}\;{\|}^{2}$$

In a training dataset including *D* instances, the cost function is defined as follows:

(13) $$\sum_{l=1}^{n_{l\;}-1}\sum_{i=1}^{s_{l\;}}\sum_{j=1}^{s_{l\;}+1}{\left(W_{ji}^{\left(l\right)}\right)}^{2}=\left[\;\frac{1}{D}\;\sum_{i=1}^{D}(\frac{1}{2}\;\|h_{w,b}\left(x^{\left(i\right)}-{\tilde{x}}^{\left(i\right)}\right){\|}^{2})\right]+\frac{\lambda }{2}\sum_{l=1}^{n_{l\;}-1}\sum_{i=1}^{s_{l\;}}\sum_{j=1}^{s_{l\;}+1}{\left(W_{ji}^{\left(l\right)}\right)}^{2}$$

where D refers to the total number of instances, *s* to the number of neurons in layer l,  λ represents the weight attenuation parameter, and the square error is the reconstruction error of each training instance.

### 3.4. Performance Measurements

The statistical analysis included the calculation of the mean square error (*MSE*), Pearson’s correlation coefficient (*R*), and the root-mean-square error (*RMSE*) to test the proposed algorithms’ efficiency in detecting Android malware. The equations of these parameters are presented below:

(14) $$MSE=\frac{1}{n}\;\sum_{i=1}^{n}{\left(y_{i,exp}-y_{i,\;pred}\;\right)}^{2}$$

(15) $$RMSE=\sqrt{\sum_{i=1}^{n}\frac{{\left(y_{i,exp}-y_{i,pred}\right)}^{2}}{n}}$$

(16) $$R\%=\frac{n\left(\sum_{i=1}^{n}y_{i,exp}\;\times_{\;}y_{i,\;pred}\right)-\left(\sum_{i=1}^{n}y_{i,exp}\right)\left(\sum_{i=1}^{n}y_{i,\;pred}\right)}{\sqrt{\left[n{\left(\sum_{i=1}^{n}y_{i,exp}\right)}^{2}-{\left(\sum_{i=1}^{n}y_{i,exp}\right)}^{2}\right]\left[n{\left(\sum_{i=1}^{n}y_{i,pred}\right)}^{2}-{\left(\sum_{i=1}^{n}y_{i,pred}\right)}^{2}\right]}}\times 100$$

(17) $$R^{2}\;bn1-\frac{\sum_{i=1}^{n}\;{\left(y_{i,\;exp}-y_{i,\;pred}\right)}^{2}}{\;\sum_{i=1}^{n}\;{\left(y_{i,\;exp}-y_{avg,\;exp}\right)}^{2}\;}$$

(18) $$Accuracy=\frac{TP+TN}{TP+FP+FN+TN}\times 100\%$$

(19) $$Specificity=\frac{TN}{TN+FP}\times 100\%$$

(20) $$Sensitivity=\frac{TP}{TP+FN}\times 100\%$$

(21) $$\mathrm{Precision}=\frac{TP}{TP+FP}\times 100\%$$

(22) $$\mathrm{Fscore}\;=\frac{2*preision\;*\;\mathrm{Sensitivity}}{preision+\mathrm{Sensitivity}}\times 100\%$$

where $y_{i,exp}$ is the experimental value of the data point *i*,$\;y_{i,pred}$ is the predicted value of the data point i, $y_{avg,exp}$ is the average of the experimental values, *R* is Pearson’s correlation coefficient, $y_{i,exp}$ are the Android network packets of the input data i, $y_{i,class}$ are the classes of Android malware and normal input data i, n is the total number of the input data, the true positive (*TP*) represents the total number of samples that are successfully classified as positive sentiment, false positive (*FP*) is the total number of samples that are incorrectly classified as negative sentiments, true negative (*TN*) denotes the total number of samples that are successfully classified as negative sentiment, and false negative (*FN*) represents the total number of samples that are incorrectly classified as positive sentiments.

## 4. Results

The investigation of the effect of the proposed models on the standard Android malware datasets was conducted using the Python programing language. The statistical analysis evaluated the results of the proposed models.

### 4.1. Splitting the Data

The datasets were divided into 70% training and 30% testing data. The random function for splitting the training and testing was proposed. The training phase was applied to fit the models using the Android malware datasets. The test phase was designed to validate the proposed models using new data. Table 3 show the datasets’ volume.

### 4.2. Experimental Environments

The platform used to detect intrusion in Android applications is presented in Table 4.

### 4.3. Model Performance

The highly efficient performance of machine learning and deep learning models guarantees the detection of Android malicious applications. The algorithms for intrusion detection were tested using two standard malware mobile datasets. The Drebin dataset contained 10,525 Android applications, and the CICAndMal2017 dataset contained 676 injections of various attack and normal packets.

#### 4.3.1. Performance of the Machine Learning Models

In this work, the SVM, KNN, and LDA models were applied to identify Android malicious packets. The SVM algorithm achieved maximum accuracy (100%) with respect to all the performance measurements in the CICAndMal2017 dataset (Table 5). However, it achieved lower accuracy (80.71%) with the Drebin dataset.

**Figure 10:**
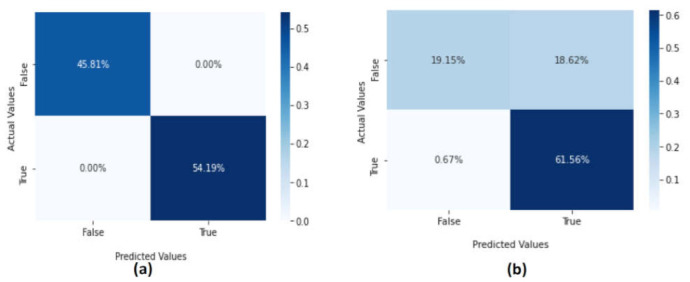
The confusion metrics of the SVM method using the (**a**) CICAndMal2017 and (**b**) Drebin datasets.

The SVM method showed the efficiency performance with the CICAndMal2017 dataset and satisfying results in the Drebin dataset. The confusion metrics of the SVM method are presented in Figure 10. In the CICAndMal2017 dataset, the percentage of the normal data classified as true negative was 45.81%, whereas the true positive represented 54.19% and were classified as malware attacks. Furthermore, the false positive and false negative data were 0, indicating that the SVM method successfully detected malicious attacks in the Drebin dataset. The confusion metrics of the SVM approach applied on the Drebin dataset were as follows: 61.56% were classified as abnormal applications, 19.15% true negatives were classified as normal applications, whereas the true positive and false negatives were 18.62% and 0.67%, respectively. We conclude that the performance of the SVM method is good since the false positive is low.

Table 6 summarize the performance of the KNN method in the detection of malware attacks in both datasets. We considered the scope of the KNN method with (k = 5). In the CICAndMal2017 dataset, the KNN method achieved high accuracy (90%), contrary to the Drebin dataset (81.57%).

**Figure 11:**
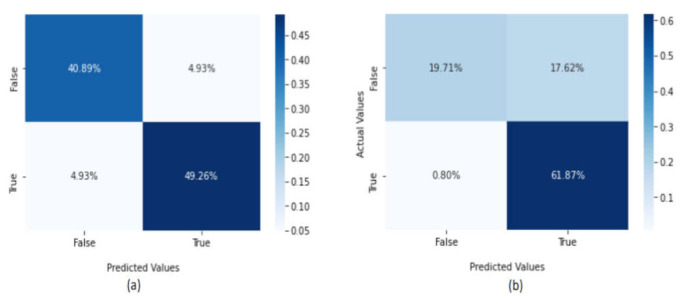
The confusion metrics of the KNN method using the (**a**) CICAndMal2017 and (**b**) Drebin datasets.

Figure 11 show the confusion metrics for the KNN method. In the CICAndMal2017 dataset, 40.89% of the dataset was classified as true negative (normal applications), 49.26% as malware, and 4.93% as false positives (normal data classified as attacks). In the Drebin dataset, the KNN method classified 61.87% of the dataset as true positives (attacks), 19.71% as true negatives (normal), and the false positives were <0.80%. Overall, the KNN method achieved higher accuracy in the CICAndMal2017 dataset than in the Drebin dataset.

The results of the LDA method are presented in Table 7. Overall, the results were not adequate due to the complexity of the network dataset. The nonlinear algorithms are not appropriate for the analysis of network datasets. The accuracy of LDA was 45.32% in the CICAndMal201 dataset, a percentage that reached 81% in the case of the Drebin dataset.

**Figure 12:**
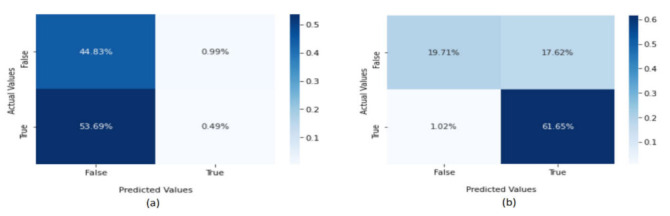
The confusion metrics for the (**a**) CICAndMal2017 and (**b**) Drebin datasets.

The confusion metrics of the LDA method are presented in Figure 12. The percentage of true positives was high (49%), whereas that of true negatives (classified as normal applications) was low (44.83%) in the CICAndMal2017 dataset. The percentage of false positives was high (53.69%), showing that the LDA model is not appropriate for this dataset. In the Drebin dataset, the confusion metrics showed that 19.15% were true negatives and 1.02% false positives, classifying normal applications as malware. Overall, the LDA method had good performance with the Drebin dataset.

#### 4.3.2. Performance of the Deep Learning Models

In this section, the results of the deep learning algorithms, namely LSTM, CNN-LSTM, and AE, are presented. The dataset was divided into 70% training and 30% test data. Table 8 show the results of the LSTM, CNN-LSTM, and AE models. The performance of the CNN-LSTM model achieved high accuracy (95.07%) compared with the LSTM and AE models in the CICAndMal2017 dataset.

**Figure 13:**
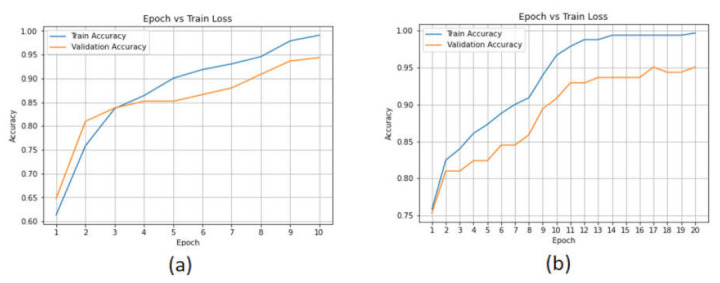
Performance of the deep learning models with the CICAndMal2017 dataset. (**a**) LSTM. (**b**) CNN-LSTM.

Figure 13 show the accuracy performance of the LSTM, CNN-LSTM, and AE algorithms using the CICAndMal2017 dataset. The performance plots show that the CNN-LSTM model achieved an accuracy of 99.9% in the training phase, and in the validation phase, the initial 75% accuracy reached 95.07%. The LSTM model achieved good performance in the training phase (99%) and the validation phase it reached 94.58%.

**Figure 14:**
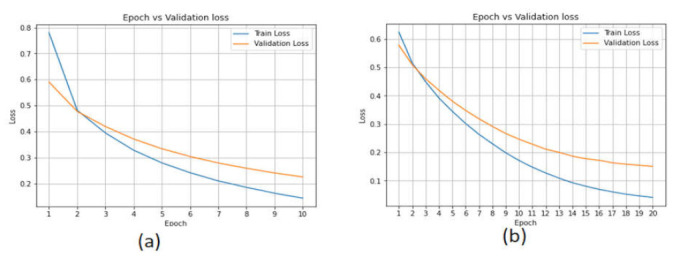
Accuracy loss of the deep learning models in the CICAndMal2017 dataset. (**a**) LSTM. (**b**) CNN-LSTM.

The binary_crossentropy method was used to calculate the accuracy loss in the training and testing phases. Figure 14 show the validation accuracy of the deep learning models. The accuracy loss of the LSTM model in the validation phase changed from 0.5 to 0.2, while in the case of the CNN-LSTM model, this changed from 0.6 to 0.2. 

Table 9 show the results of the LSTM, CNN-LSTM, and AE models using the Drebin dataset. The LSTM model achieved high accuracy (99.40%). Furthermore, the CNN-LSTM model showed high accuracy of 97.20%, and the performance of the AE model was satisfying.

**Figure 15:**
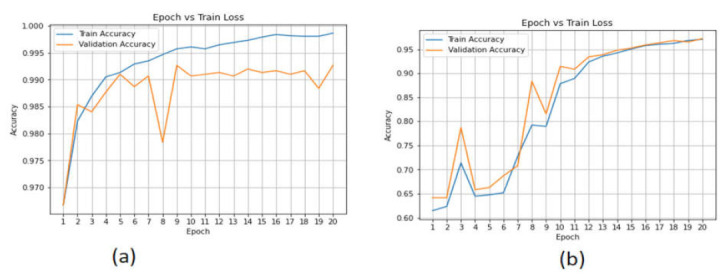
Performance of the deep learning models in the CICAndMal2017 dataset. (**a**) LSTM. (**b**) CNN-LSTM.

Figure 15 show the accuracy performance of the deep learning models. The validation accuracy of the LSTM model started from 97% and reached 99.40% with 20 Epochs. The LSTM model in the training phase achieved an accuracy of 100%. The performance of the CNN-LSTM model was 97.20% in the validation phase.

**Figure 16:**
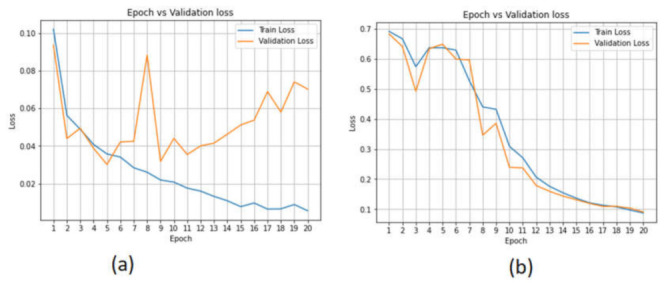
Accuracy loss of the deep learning models in the CICAndMal2017 dataset. (**a**) LSTM. (**b**) CNN-LSTM.

Figure 16 show the validation loss of the deep learning models. In the LSTM model, the validation loss changed from 0.10 to 0.7, whereas for the CNN-LSTM model, it changed from 0.7 to 0.1 with 20 Epoch.

**Figure 17:**
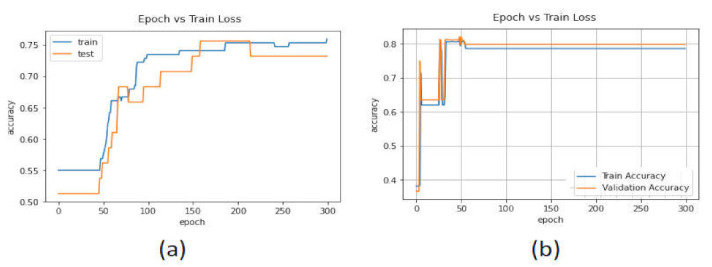
Accuracy of the AE model in the (**a**) CICAndMal2017 and (**b**) Drebin datasets.

The accuracy performance of the AE model using the CICAndMal2017 and Drebin datasets is presented in Figure 17. The performance of AE was not satisfying, with the accuracy in the training phase being 79% and in the validation phase 75.79% for the CICAndMal2017 dataset. For the Drebin dataset, the accuracy in the validation phase was 56%. The accuracy percentage of the LSTM and CNN-LSTM models outperformed the AE model.

**Figure 18:**
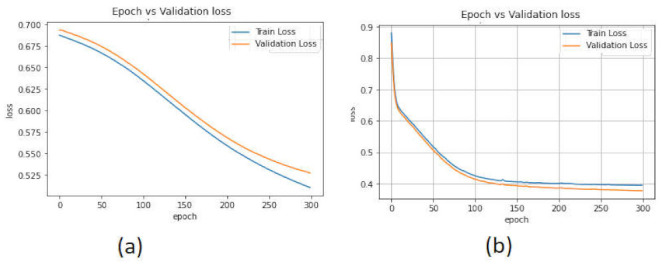
Accuracy loss of the autoencoder model in the (**a**) CICAndMal2017 and (**b**) Drebin datasets.

Figure 18 display the accuracy loss of the AE model in both datasets. The accuracy loss was high (from 0.70 to 0.55) for the CICAndMal2017 dataset. Furthermore, the validation loss changed from 0.9 to 0.4 in the case of the Drebin dataset. Overall, the validation loss of the AE model was high; therefore, the AE model’s performance is not appropriate for the detection of Android malicious attacks.

### 4.4. Sensitivity Analysis

Sensitivity analysis is an approach used to measure the influence of uncertainties of the input data variables. Analyzing the input data is very useful in extracting the patterns from the dataset. The Pearson’s correlation coefficient was applied to find the correlation between the input features and the classes. Some features had significant relationships between the classes (normal and attacks) [70,71]. 

**Figure 19:**
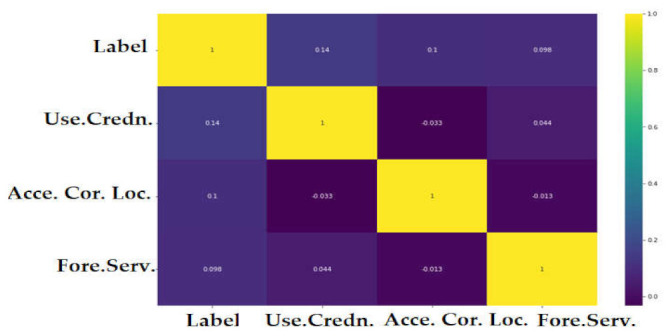
The correlation coefficient results using the CICAndMal2017 dataset.

**Figure 20:**
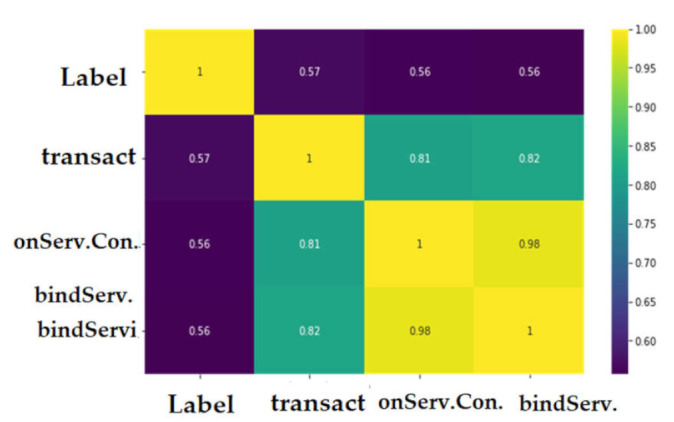
The correlation coefficient for the Drebin dataset.

We selected the features that had a relationship >50% between the class. Figure 19 show the features that have a significant correlation with the classes variables in the CICAndMal2017 dataset. We considered four features with correlation >50%. The correlation coefficient results for the Drebin dataset are presented in Figure 20. It was observed that the Drebin dataset revealed a strong correlation between classes, while in the CICAndMal2017 dataset, they were <50%.

We applied the statistical metrics mean absolute error (MAE), MSE, RMSE, and R^2^ to identify the prediction error between the target class and the predicted values. The prediction error of the machine learning algorithms is presented in Table 10. The SVM algorithm displayed fewer prediction errors, and the R^2^ between the predicted values and the target values was 100% for the CICAndMal2017dataset. The KNN method showed fewer prediction errors (MSE = 0.1842), and the relationship between the predicted and target values was 33.35%.

Table 11 show the prediction potential of the SVM, KNN, and LDA methods. The prediction performance of the KNN method was R^2^ = 33.35, achieving the best correlation between the predicted and target values in the Drebin dataset. Overall, the prediction results of the machine learning algorithms were satisfactory.

The prediction errors of the deep learning algorithms are summarized in Table 12. The LSTM model achieved lower prediction levels (MSE = 0.0054), and the correlation between the predicted and target values was 88.25% in the CICAndMal2017 dataset. In the Drebin dataset, the LSTM model showed lower prediction levels (MSE = 0.0059) and high correlation (R^2^ = 97.39%). The prediction performance of LSTM was good in both datasets.

## 5. Discussion

With rapidly developing technology, the use of smartphones with new features and associated Android applications has increased. Statista reported that 1.3 billion smartphones will be used by 2023. This also brings challenges for the researchers and developers of security mechanisms for these applications, originating in the new complexities and vulnerabilities of the Android applications that hackers can quickly exploit.

Considering that Android applications of digital e-commerce, e-business, savings, and online banking are associated with confidential and appreciated information communicated within the mobile network, it is important to evaluate the application data in terms of accomplishing proper security. Machine and deep learning algorithms are used to monitor the detection of malicious attacks against Android applications to ensure that security openings do not occur within this network. The present research contributes to the area of cybersecurity by developing a system based on machine learning and deep learning algorithms to detect anomalies in signature databases, thus, permitting the system to detect unknown attacks. 

As we know, the network has a very complex format; in this study, nonlinear models were proposed to achieve high accuracy, whereas linear, namely LDA and KNN, models achieved slightly worse performance. The accuracy performance of LDA was 45.32% in the CICAndMal2017 dataset, and the accuracy performance improved to 81.35% using the Drebin dataset. It was observed that the KNN model achieved little accuracy, 81.57%, using the Drebin dataset. We observed that the LDA and KNN algorithms are not appropriate for detecting Android malware. In deep learning models, the AE mode results were not satisfactory for detecting the mobile attacks. The AE achieved 75.79% and 56.65% with respect to the CICAndMal2017 and Drebin datasets. The AE is composed of the encoder and decoder models; the encoder compresses the input data, whereas the decoder is used to recreate the input from the encoder. Overall, we observed that these models did not achieve good results due to the research datasets being binary data. 

Furthermore, using the support vector machine, LSTM and CNN-LSTM algorithms achieved high accuracy performance for developing an appropriate system that can support the security of smartphones against malware. Two standard datasets were used. The SVM model achieved an accuracy of 100% using the CICAndMal2017 dataset and the LSTM algorithm achieved 99.40% using the Drebin dataset.

**Figure 21:**
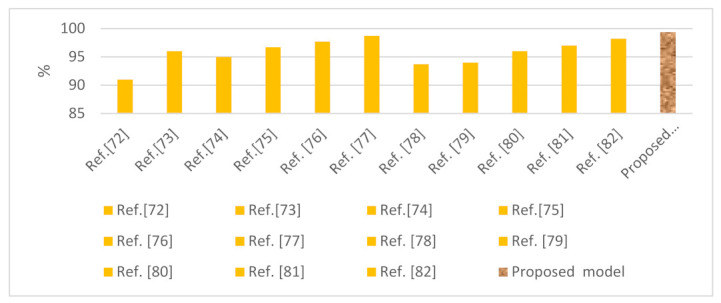
Comparative performance of the proposed system against existing systems in the detection of malware against Android applications using the Drebin dataset.

Our system was compared with existing systems of machine learning and deep learning models that detect malware for the security of Android applications. The mechanism of the proposed system is based on the pattern of dataset behavior for detecting the attacks. The LSTM model had an accuracy of 99.40% in the case of the Drebin dataset, indicating that it is a robust model to handle Android security vulnerabilities. Recently, by employing a CNN model on an Android platform, the system was found to achieve high accuracy; however, our system is more accurate against all systems. Table 13 show the results of our system against existing security systems using the same dataset. The graphic representation of our system and other existing systems’ results with respect to the accuracy metrics is presented in Figure 21. Overall, the system we propose achieved the highest accuracy. 

**Figure 22:**
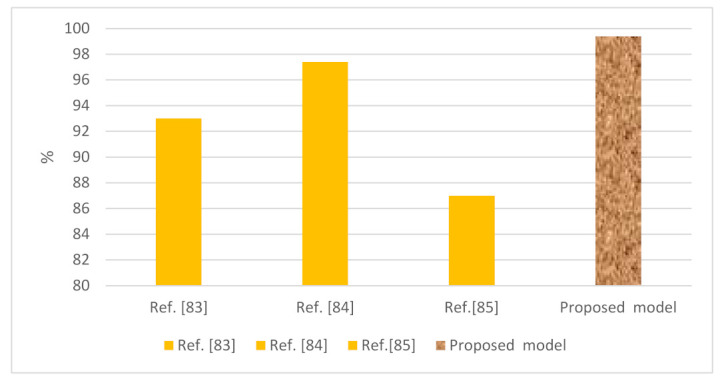
Comparative performance of the proposed system against existing systems in the detection of malware against Android applications using different datasets.

Table 14 display the results of the proposed system and other existing Android cybersecurity systems that use the machine and deep learning algorithms applied to different Android datasets. To confirm the results of the proposed system against other Android security systems, we compared recent systems’ results with ours, with the latter achieving high accuracy. The graphic representation of these results is presented in Figure 22.

## 6. Conclusions

Smartphones are becoming more and more popular, constituting a profitable target for hackers due to their susceptibility to security breaches. Android is an open gate for attackers who exploit it with malicious applications, benefiting from the system’s security flaws. An emerging method for signature-based malicious attack detection is the antivirus applications against new malware, created with AI, machine learning, and deep learning algorithms that predict malware. In this study, a security system was built and designed based on the support vector machine (SVM), k-nearest neighbors (KNN), linear discriminant analysis (LDA), long short-term memory (LSTM), convolution neural network-long short-term memory (CNN-LSTM), and autoencoder algorithms. According to the promising results of the present research, the following conclusions can be drawn:

The proposed system was evaluated and examined using two standard Android malware applications datasets: CICAndMal2017 and Drebin. The SVM, KNN, and LDA methods proved to be efficient machine learning algorithms and successfully detected malware, with SVM being the most effective. The LSTM and CNN-LSTM models are proposed to detect malicious applications, with the LSTM model being more efficient for developing Android security. Sensitive analysis examining the metrics MSE, RMSE, and R^2^ revealed the errors between the predicted output and the target values in the validation phase. The LSTM and CNN-LSTM algorithms achieved fewer prediction errors in the Drebin dataset, while the SVM method was more effective in the case of the CICAndMal2017 dataset. The validation phase results of the machine learning and deep learning methods were satisfying, with the LSTM and SVM models achieving superior performance. The results of the present study were compared with recent research findings, confirming the robustness and effectiveness of our results. We implemented machine learning and deep learning algorithms and experimented with them to obtain optimal malware detection. Both of the proposed classifiers achieved good accuracy, but the LSTM accuracy was 99.40%, indicating it can outperform other state-of-the-art models.

## Figures and Tables

**Table 1:** Parameters of the LSTM model.

Parameters	Values
Kernel size	4
Max pooling size	4
Drop out	0.50
Fully connected layer	32
Activation function	Relu
Optimizer	RSMprop
Epochs	10, 20
Batch size	20

**Table 2:** Parameters of the CNN-LSTM model.

Parameters	Values
Kernel size	4
Max pooling size	4
Drop out	0.50
Fully connected layer	32
Activation function	Relu
Optimizer	RSMprop
Epochs	20
Batch size	150

**Table 3:** Volume of datasets.

Datasets	Total Volume	Training	Testing
CICAndMal2017	676	473	203
Drebin	15,031	10,521	4510

**Table 4:** Environment requirements of the proposed model.

Hardware	Software
RAM size 8 GB	Python Version 3.6
C.P.U.	Numpy Version 1.18.1
	TensorFlow library Version 2.10
	Keras library Version 2.3.1
	Matplotlib Version 3.2.0
	NumPy library Version 1.01

**Table 5:** Results of the SVM method.

	CICAndMal2017 Dataset		
Metrics	Precision (%)	Recall (%)	F1-score (%)
Normal	100	100	100
Attacks	100	100	100
Accuracy	100		
Weighted average	100	100	100
	Drebin dataset		
Metrics	Precision (%)	Recall (%)	F1-score (%)
Normal	0.97	0.51	0.67
Attacks	0.77	0.99	0.86
Accuracy	80.71		
Weighted average	0.84	0.81	0.79

**Table 6:** Results of KNN algorithm.

	CICAndMal2017		
Metrics	Precision (%)	Recall (%)	F1-score (%)
Normal	0.89	0.89	0.89
Attacks	0.91	0.91	0.91
Accuracy	0.90		
Weighted average	0.90	0.90	0.90
	Drebin dataset		
Metrics	Precision (%)	Recall (%)	F1-score (%)
Normal	0.96	0.53	0.68
Attacks	0.78	0.99	0.87
Accuracy	81.57		
Weighted average	0.85	0.82	0.80

**Table 7:** Results of the LDA method.

CICAndMal201			
Metrics	Precision (%)	Recall (%)	F1-Score (%)
Normal	0.46	0.98	0.62
Attacks	0.33	0.01	0.02
Accuracy	45.32		
Weighted average	0.39	0.45	0.29
Drebin Dataset			
Metrics	Precision (%)	Recall (%)	F1-score (%)
Normal	0.95	0.53	0.68
Attacks	0.78	0.98	0.87
Accuracy		81.35	
Weighted average	84	0.81	0.82

**Table 8:** Results of the deep learning algorithms in the CICAndMal2017 dataset.

Models	Loss	Accuracy (%)	Precision (%)	Recall (%)	F1 Score (%)
LSTM	0.20	94.58	95.41	94.54	94.97
CNN-LSTM	0.16	95.07	97.16	93.63	95.53
AE	1.43	75.79	92.15	66.78	77.44

**Table 9:** Results of the deep learning models using the Drebin dataset.

Models	Loss	Accuracy (%)	Precision (%)	Recall (%)	F1 Score (%)
LSTM	0.05	99.40	99.32	99.74	99.53
AE	3.65	56.65	41.18	65.71	51.11
CNN-LSTM	0.09	97.20	97.72	97.92	97.82

**Table 10:** Statistical analysis of the machine learning algorithms’ results using the CICAndMal2017 dataset.

Models	MAE	MSE	RMSE	R^2^ (%)
SVM	0.00	0.0	0.0	100
KNN	0.0985	0.09852	0.313	63.31
LDA	0.429	0.4189	0.647	53.68

**Table 11:** Statistical analysis of the machine learning models using the Drebin dataset.

Models	MAE	MSE	RMSE	R^2^ (%)
SVM	0.1915	0.1915	0.437	31.57
KNN	0.1842	0.1842	0.429	33.35
LDA	0.1864	0.1864	0.431	32.09
SVM	0.1915	0.1915	0.437	31.57

**Table 12:** Statistical analysis of the deep learning models.

	CICAndMal2017 Dataset			
Models	MAE	MSE	RMSE	R^2^ (%)
LSTM model	0.0054	0.0541	0.232	88.25
Autoencoder model	0.339	0.339	0.5830	31.74
CNN-LSTM	0.049	0.049	0.221	80.31
Drebin dataset				
Models	MAE	MSE	RMSE	R^2^ (%)
LSTM model	0.0059	0.0059	0.077	97.39
Autoencoder model	0.2425	0.2279	0.177	17.91
CNN-LSTM	0.027	0.027	0.1671	87.84

**Table 13:** Results of the proposed system against existing security systems using the Drebin dataset.

Reference	Year	Datasets	Model	Accuracy (%)
Ref. [72]	2021	Drebin	CNN	91
Ref. [73]	2018	Drebin	RF, J.48, NB, Simple Logistic, BayesNet TAN, BayesNet K2, SMO PolyKernel, IBK, SMO NPolyKernel	88–96
Ref. [74]	2021	Drebin	CBR, SVM, DT	95
Ref. [75]	2019	Drebin	Random forest tree	96.7
Ref. [76]	2018	Drebin	DT	97.7
Ref. [77]	2019	Drebin	RF with 1000 decision trees	98.7
Ref. [78]	2019	Drebin	SVM	93.7
Ref. [79]	2019	Drebin	Random forest tree	94
Ref. [80]	2019	Drebin	Random forest tree	96
Ref. [81]	2016	Drebin	Random forest tree	97
Ref. [82]	2021	Drebin	CNN	98.2
Proposed model	2022	Drebin	LSTM CNN-LSTM	99.40 97.82

**Table 14:** Results of the proposed system against existing security systems using different Andriod datasets.

Reference	Year	Datasets	Model	Accuracy (%)
Ref. [83]	2019	MalGenome, Kaggle, Androguard	Random forest tree	93
Ref. [84]	2018	Google Play, VirusShare, MassVet	LSTM	97.4
Ref. [85]	2017	Genome, IntelSecurity, MacAfee, Google Play	Deep CNN	87
	2022	Drebin	LSTM CNN-LSTM	99.40 97.82

## Data Availability

The data presented in this study are available here: https://www.kaggle.com/saurabhshahane/Android-permission-dataset; https://www.kaggle.com/shashwatwork/android-malware-dataset-for-machine-learning (accessed on 25 Novmber 2021).
